# Co-locating public mental health services in communities: a realist evaluation: Shamini Gnani

**DOI:** 10.1093/eurpub/ckac130.215

**Published:** 2022-10-25

**Authors:** C Baskin, F Duncan, E Oliver, G Samuel, A Adams, S Gnani

**Affiliations:** 1 Department of Primary Care and Public Health, Imperial College London, London, UK; 2 Department of Sport and Exercise Sciences, Durham University, Durham, UK; 3 McPin Foundation, London, UK; 4 Population Health Sciences Institute, Newcastle University, Newcastle, UK

## Abstract

**Background:**

Public mental health (PMH) services address social determinants of mental health, such as poverty, poor housing, and job insecurity. Austerity and welfare reform in the UK has led to cuts to social and welfare support, increasing poor mental health and widening inequalities, exacerbated by COVID-19. State health services lack capacity to tackle social issues that contribute to a large proportion of expressed mental health need. Co-locating PMH services within community spaces is a potential solution to increase early access and improve quality of services. Using a realist evaluation, we sought to develop the theory on how community co-location affects PMH outcomes, who this works best for, and how this is impacted by the context of delivery.

**Methods:**

We collected data from service-users and service-providers at six case study sites across England, UK, using interviews (n = 62), four focus groups (n = 40) and two stakeholder workshops (n = 19).

**Results:**

We identified four overarching theories. First, community providers do not operate under the same limits as state services allowing them the flexibility and time to build trust and ongoing relationships with service users. Second, the ethos and culture of services is to empower users to access help and be independent. Third, accessing support from a shared local space allows a coordinated and holistic response reducing barriers such as distance, cost, and anxiety. Four, as they are recreational services and spaces for access by all with no predefined/required level of need they are better at promoting wellbeing and primary prevention.

**Conclusions:**

Community co-location of PMH services can strengthen the overall mental health system by widening reach to people vulnerable to poor mental health and enabling earlier intervention on associated social determinants. This has potential to reduce mental health inequalities and demand on the state health system.

**Key messages:**

- Community co-location of PMH services can provide early and holistic support for complex social issues.
- There is potential to support the state health system by alleviating demand for help with ‘non-health’ issues.

